# Supplementary material for: Microbiota Transplantation in Day-Old Broiler Chickens Ameliorates Necrotic Enteritis via Modulation of the Intestinal Microbiota and Host Immune Responses
Source: Pathogens. 2022 Aug 26;11(9):972. doi: 10.3390/pathogens11090972 (PMC9503007; doi:10.3390/pathogens11090972)
Supplement: Supplementary file 1 [file pathogens-11-00972-s001.zip › Pathogens-1878652_Supplemental.pdf]

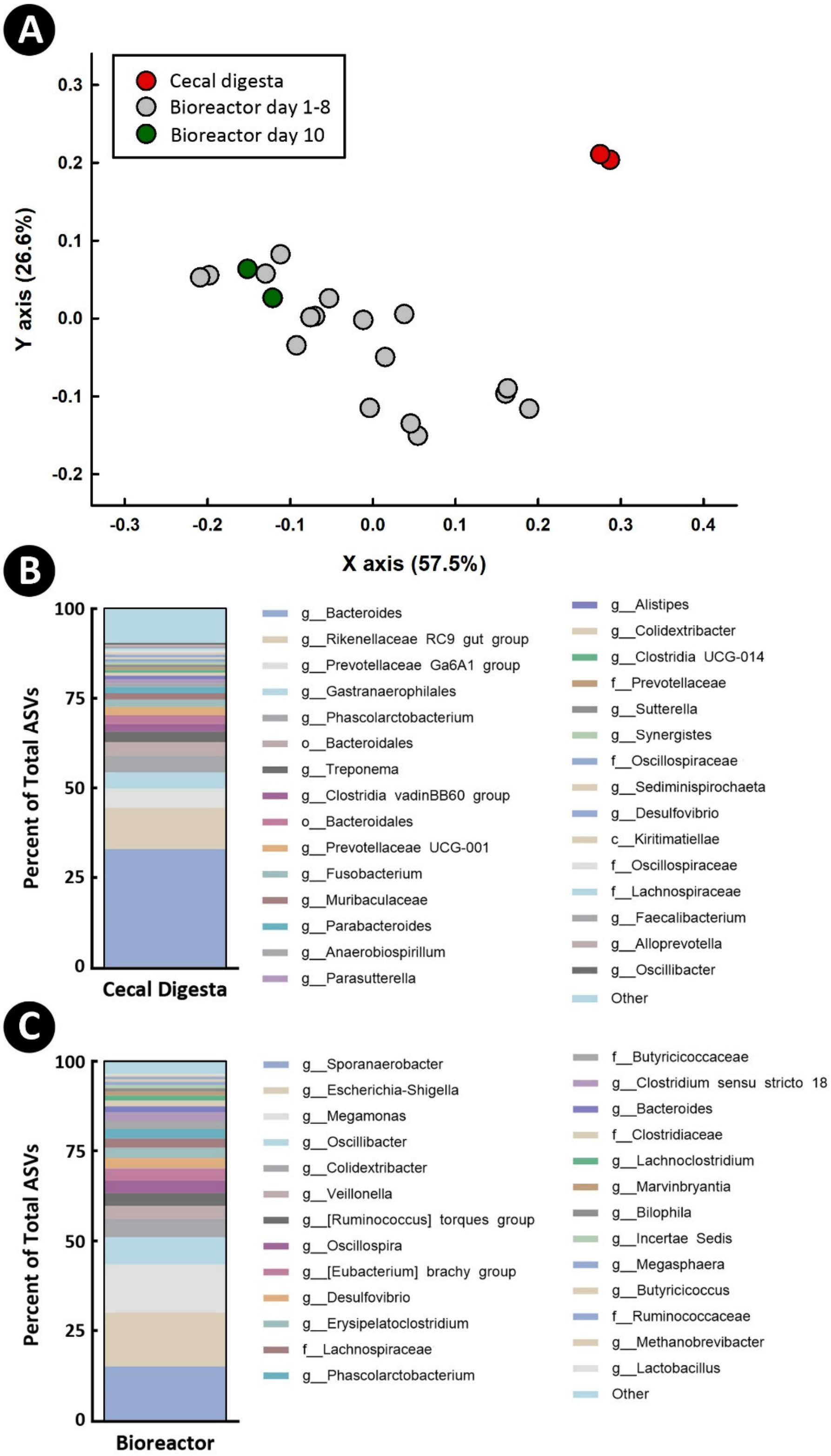

Figure S1. Bacterial communities in the cecal digesta of donor broilers and within bioreactors. (A) Weighted UniFrac β-diversity of bacterial communities within bioreactors over time. (B) Composition of bacteria in cecal digesta obtained from adult male broiler donors. (C) Composition of bacteria within the bioreactor on day 10. Taxonomic designations are: c_, class; o_, order; f_, family; and g_, genus.

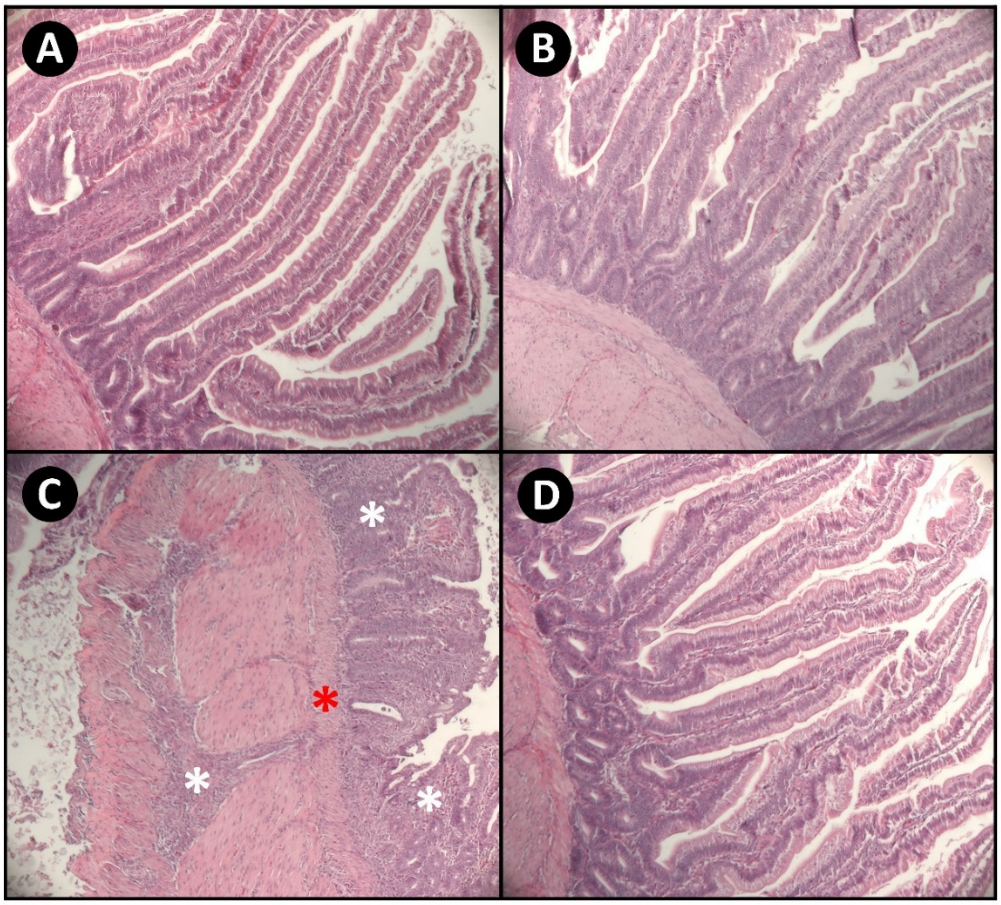

Figure S2. Representative micrographs of the jejunum of broilers. At 1-day post-hatch, birds were orally administered a microbiota transplant (MT) originating from adult broiler breeder birds and propagated within bioreactors, or medium alone. On days 12 and 13 post-hatch, birds were orally administered 1-2 x 10^8 colony forming units of *C. perfringens*, the incitant of necrotic enteritis (NE) (i.e. NE and MT + NE treatments) or buffer alone (i.e. Control and MT treatments). (A) Control treatment. (B) MT treatment. (C) NE treatment. (D) MT + NE treatment. White asterisks denote areas of substantive immune cell infiltration into the lamina propria with a high degree of villar atrophy and villar fusion. The red asterisk denotes infiltration of immune cells into the jejunal musculature.

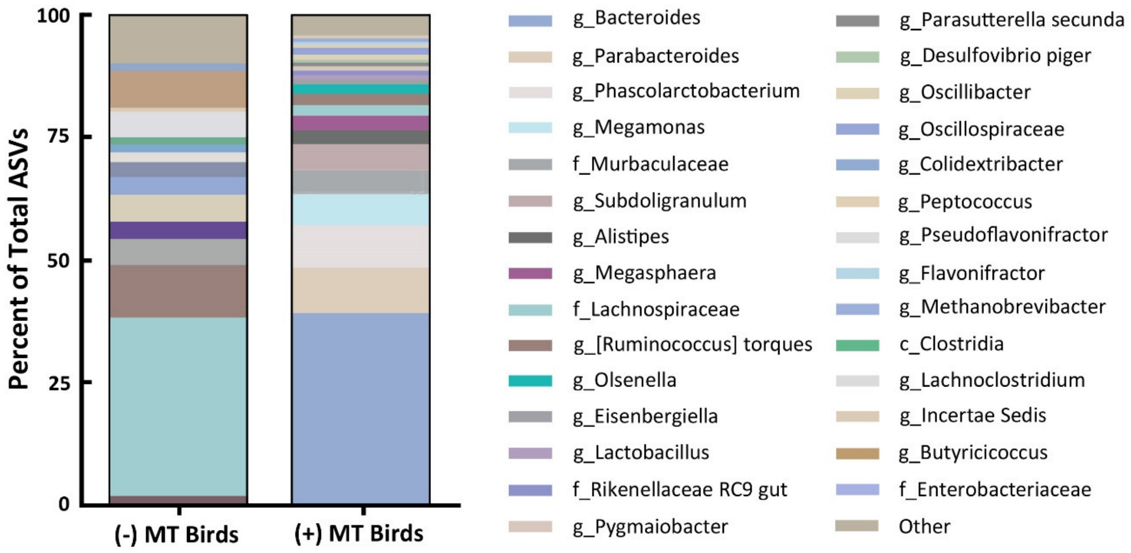

Figure S3. Composition of bacteria in ceca of broilers ± the administration of a microbiota transplant (MT). At 1-d post-hatch, birds were orally administered the transplant originating from the ceca of healthy adult broiler breeder birds that was propagated within bioreactors, or medium alone. Taxonomic designations are: c_, class; f_, family; and g_, genus. Twelve replicate birds were analyzed per MT treatment.

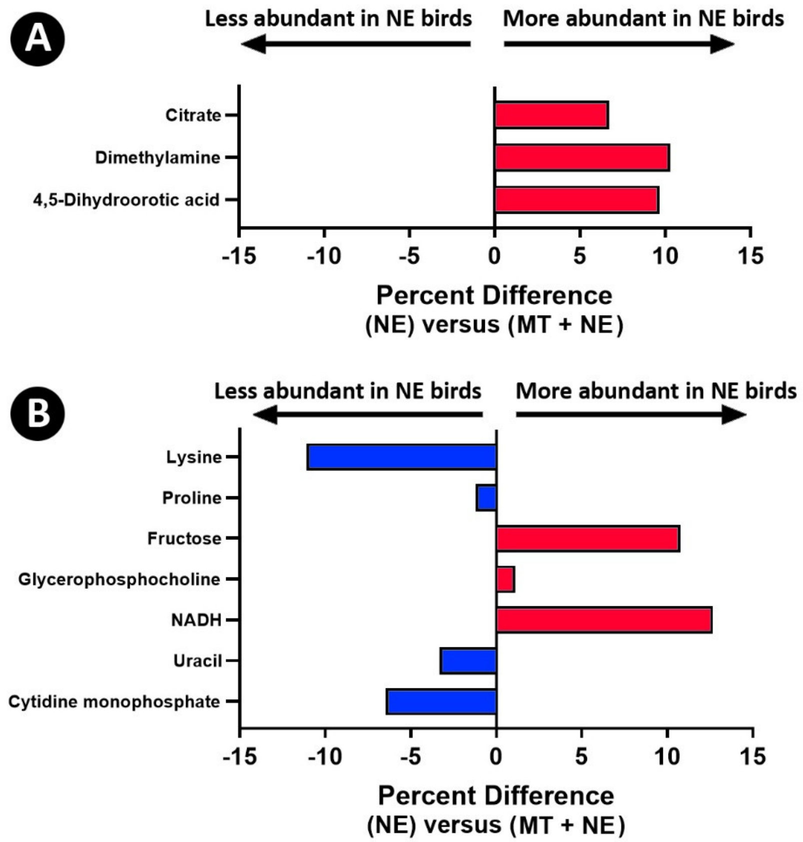

Figure S4. Metabolites that were differentially abundant within jejunal digesta (A) and tissue (B) of broilers. At 1-day post-hatch, birds were orally administered a microbiota transplant (MT) originating from adult broiler breeder birds and propagated within bioreactors, or medium alone. On days 12 and 13 post-hatch, birds were orally administered 1-2 x 10^8 colony forming units of *Clostridium perfringens*, the incitant of necrotic enteritis (NE) (i.e. NE and MT + NE treatments). Six replicate birds were analyzed per treatment.

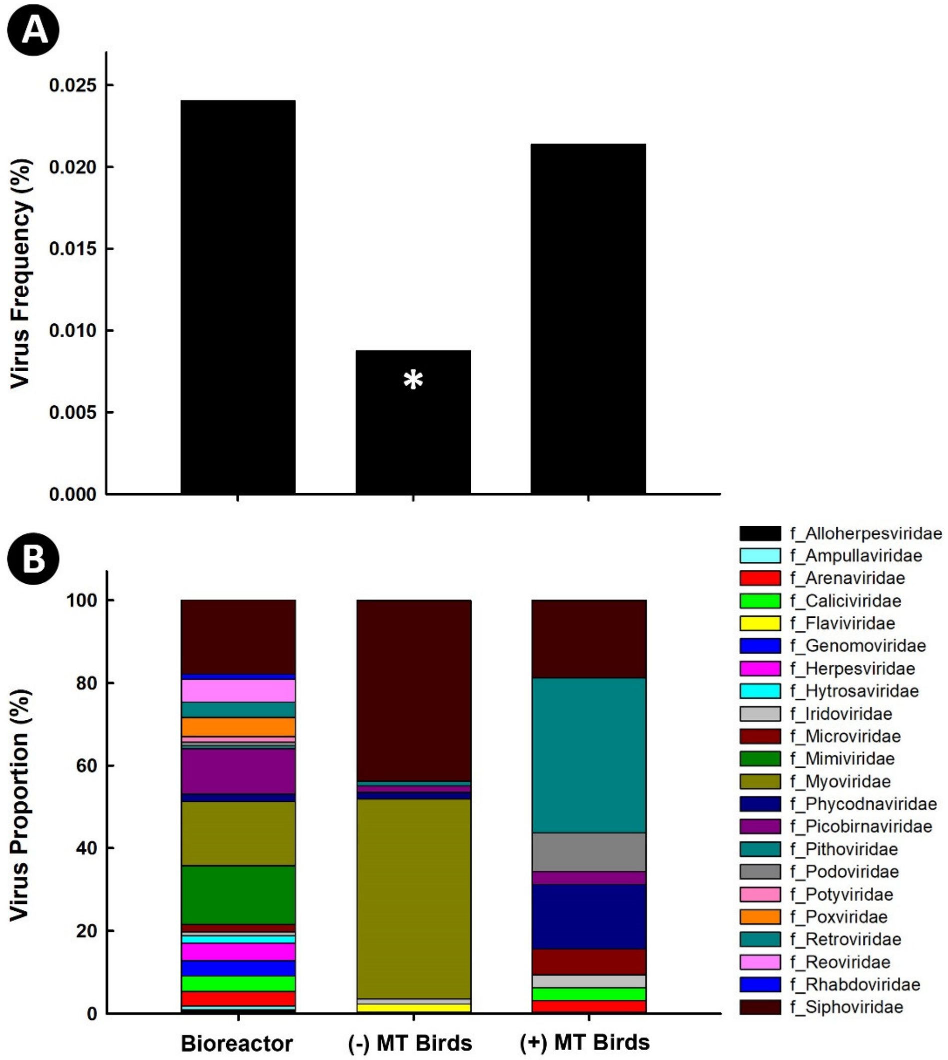

Figure S5. Frequencies of viruses in the bioreactor and feces obtained from broilers ± the administration of a microbiota transplant (MT). At 1-d post-hatch, birds were orally administered a MT originating from the ceca of healthy adult broiler breeder birds that was propagated within bioreactors, or medium alone. Overall, virus frequency was based on total microbial read counts within the samples (i.e. eukaryotes, prokaryotes, and RNA and DNA viruses). (A) Total viral read counts (%). The asterisk denotes a substantially lower total abundance of viral sequences in birds not administered a MT. (B) Proportion of viral families (%). Taxonomic designations are: f_, family. Twelve replicate birds were analyzed per MT treatment.

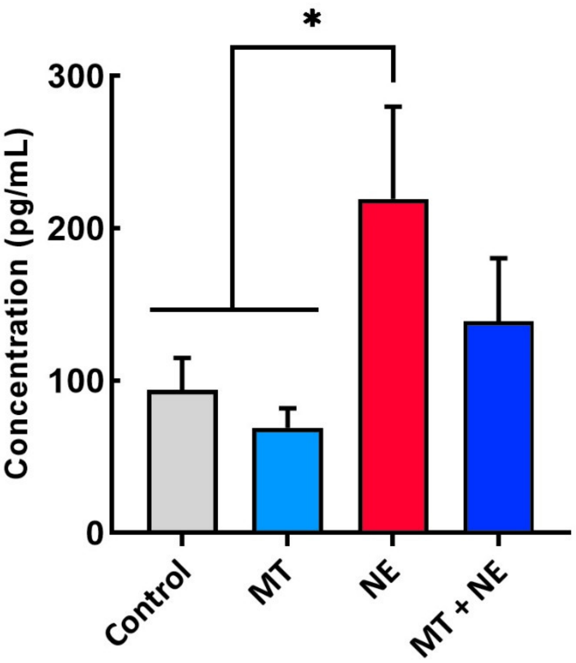

Figure S6. Corticosterone concentrations in serum of broilers. At 1-day post-hatch, birds were orally administered a microbiota transplant (MT) originating from adult broiler breeder birds and propagated within bioreactors, or medium alone. On days 12 and 13 post-hatch, birds were orally administered 1-2 x 10^8 colony forming units of *Clostridium perfringens*, the incitant of necrotic enteritis (NE) (i.e. NE and MT + NE treatments) or buffer alone (i.e. Control and MT treatments). All birds were administered corticosterone in feed at a concentration of 20 mg/kg feed commencing on day 11 post-hatch. The asterisk denotes differences ($p < 0.050$) among treatments. Six replicate birds were analyzed per treatment.

Table S1. Composition of broiler starter and grower diets.

Ingredient	Starter (0 to 10 d-of-age) %	Grower (11 to 14 d-of-age) %
Corn	49.53	54.68
Soybean meal	43.06	37.31
Canola oil	2.39	3.26
Salt	0.51	0.52
Limestone	1.52	1.41
Dicalcium phosphate	1.26	1.09
Magnesium oxide	0.10	0.15
L-Lysine HCl	0.11	0.12
D,L-Methionine	0.37	0.33
L-Threonine	0.15	0.13
Vitamin premix[1]	0.50	0.50
Choline premix	0.50	0.50

[1]Corticosterone was mixed in the vitamin premix within the grower diet and administered to birds challenged with necrotic enteritis (NE) at a dose of 20 mg/kg of feed ± administration of a microbiota transplant (MT) (i.e. NE and MT + NE treatments).

Table S2. List of primer sequences for relative mRNA quantification.

Target Gene	Target Abbreviation	Sequence (5′ to 3′) Forward	Reverse	Ta (°C)	Source
Interleukin 1β	*IL1B*	TGCCTGCAGAAGAAGCCTCG	CTCCGCAGCAGTTTGGTCAT	58	Zaytsoff et al. [1]
Interleukin 17A	*IL17A*	AGATGCTGGATGCCTAACCC	GTGGTCCTCATCGATCCTGTAA	58	Zaytsoff et al. [2]
Interleukin 2	*IL2*	TAACTGGGACACTGCCATGA	GATAGAGATGCTCCATAAGCTGT	56	Zaytsoff et al. [1]
Interleukin 22	*IL22*	GGAATCGCACCTACACCTTG	GCGGTTGTTCTCCCTGATGT	58	This Study
Transforming Growth Factor β2	*TGFB2*	CCATCTACAACAGCACCAGGG	TAGCTTGGTGGGATGGCATTTTC	58	Zaytsoff et al. [1]
Toll-like Receptor 2A	*TLR2A*	CAGCACAAGAGGCGTTCA	AACATTTTGGTGTAGCTGAGATG	56	Zaytsoff et al. [1]
Mucin 2B	*MUC2B*	ATTGTGGTAACACCAACATTCATC	CTTTATAATGTCAGCACCAACTTCTC	56	Jiang et al. [3]
Mucin 5AC	*MUC5AC*	TCCACCAGCTTCCAAATCCC	GGGGTTGCCAGCCTTTACTT	58	Zaytsoff et al. [1]
Cathelicidin 1	*CATH1*	GCTGTGGACTCCTACAACCAAC	GGAGTCCACGCAGGTGACATC	55	Achanta et al. [4]
Avian β-defensin 6	*AvBD6*	AAAATCTTGCTGTGTGAGGAAC	CATTTGGTAGTTGCAGGCAG	55	This Study
Tight Junction Protein 1	*TJP1*	AGCCCCTTGGTAATGTGTGG	CCAGGTTTTGGGGTCACAGT	56	This Study
Claudin 3	*CLD3*	GGGATTTCTACAACCCGCTG	CTTGTCGTAGCTGGTAACGG	58	Zaytsoff et al. [1]

References

1. Zaytsoff, S.J.M.; Lyons, S.M.; Garner, A.M.; Uwiera, R.R.E.; Zandberg, W.F.; Abbott, D.W.; Inglis, G.D. Host responses to *Clostridium perfringens* challenge in a chicken model of chronic stress. *Gut Pathog* **2020**, *12*, 24.
2. Zaytsoff, S.J.M.; Boras, V.F.; Uwiera, R.R.E.; Inglis, G.D. A stress-induced model of acute necrotic enteritis in broiler chickens using dietary corticosterone administration. *Poultr Sci* **2022**, *101*, 101726.
3. Jiang, Z.; Applegate, T.J.; Lossie, A.C. Cloning, annotation and developmental expression of the chicken intestinal muc2 gene. *PLoS One* **2013**, *8*, e53781.
4. Achanta, M.; Sunkara, L.T.; Dai, G.; Bommineni, Y.R.; Jiang, W.; Zhang, G. Tissue expression and developmental regulation of chicken cathelicidin antimicrobial peptides. *J Anim Sci Biotechnol* **2012**, *3*, 15.
